# Supplementary material for: Development and Evaluation of Molecular Pen-Side Assays without Prior RNA Extraction for Peste des Petits Ruminants (PPR) and Foot and Mouth Disease (FMD)
Source: Viruses. 2022 Apr 17;14(4):835. doi: 10.3390/v14040835 (PMC9026347; doi:10.3390/v14040835)
Supplement: Supplementary file 1 [file viruses-14-00835-s001.zip › viruses-1601675-supplementary.pdf]

Supplementary Materials: The following materials are available online at ……..

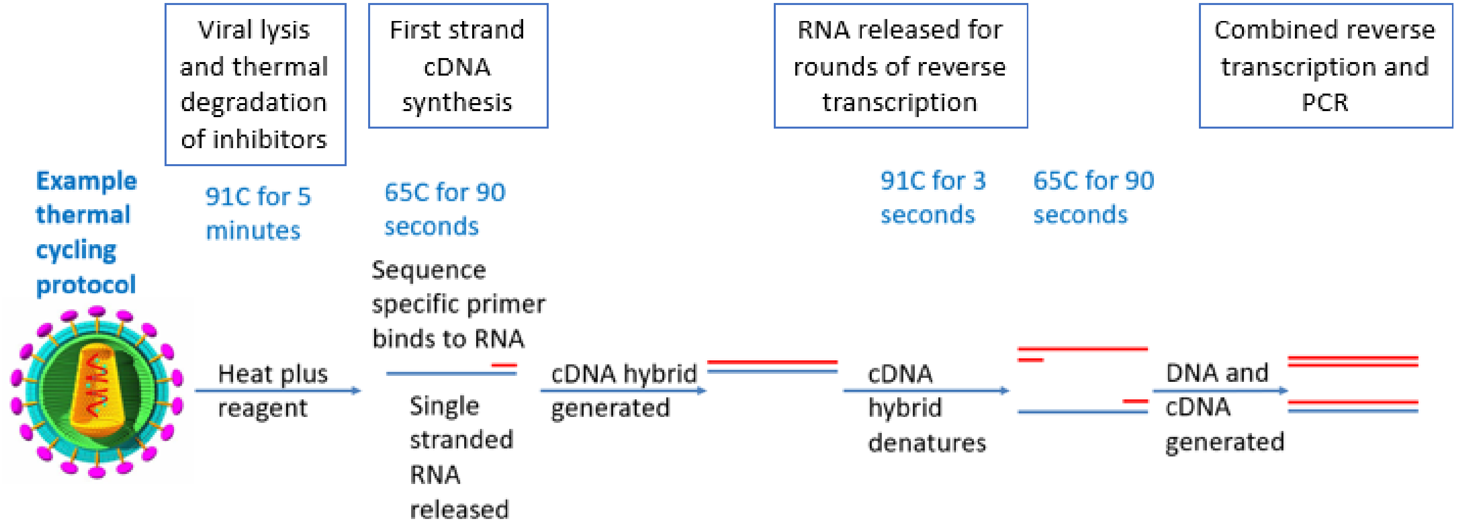

Supplementary Figure S1. Schematic representation of the XF1 reagent methodology used in this study, showing that the reagent is able to lyse target virions, rendering them amplifiable and then perform multiple rounds of reverse transcription.

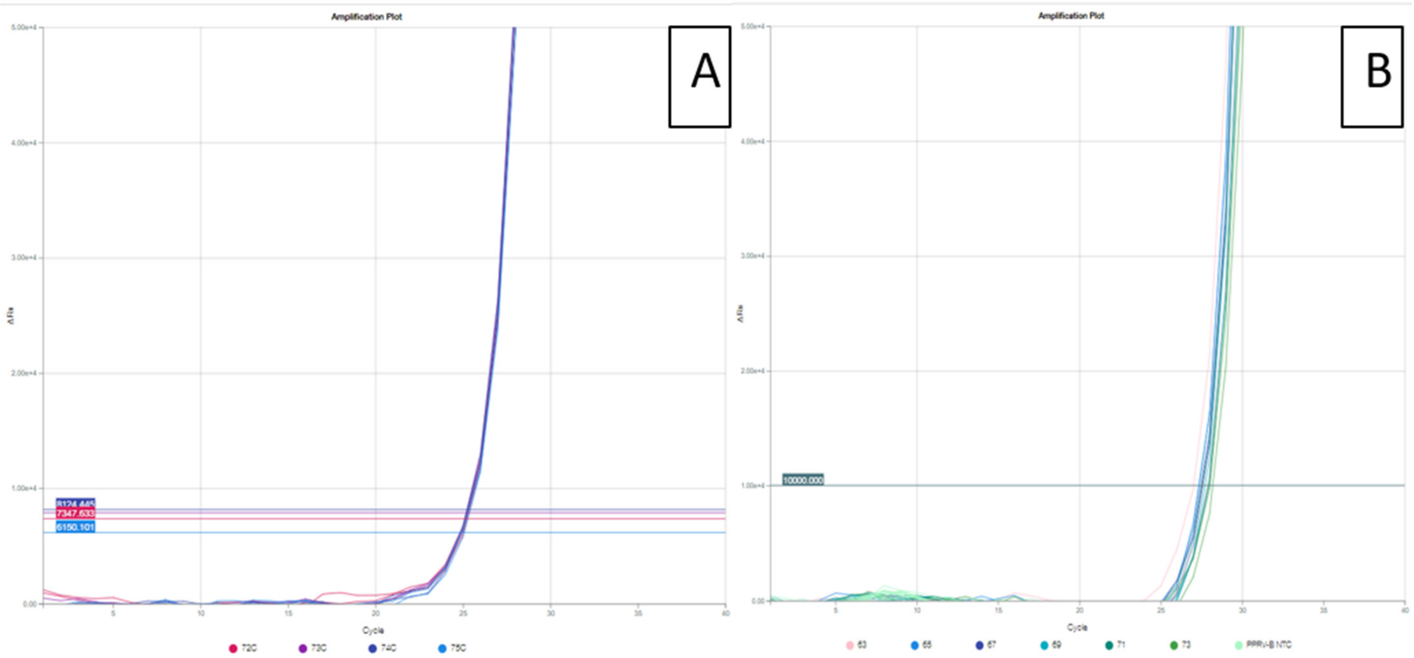

Supplementary Figure S2. Demonstration of high temperature reverse transcription for FMD (A) and PPR (B) assays -showing consistent RT activity obtained across a wide range of temperatures upon addition of 50 copies of IVT RNA to each of the two assays. For the FMD assay (A), the optimal range was 72⁰C to 75⁰C (shown). For the PPR assay (B), the optimal range was wider at 63⁰C to 73⁰C (shown).

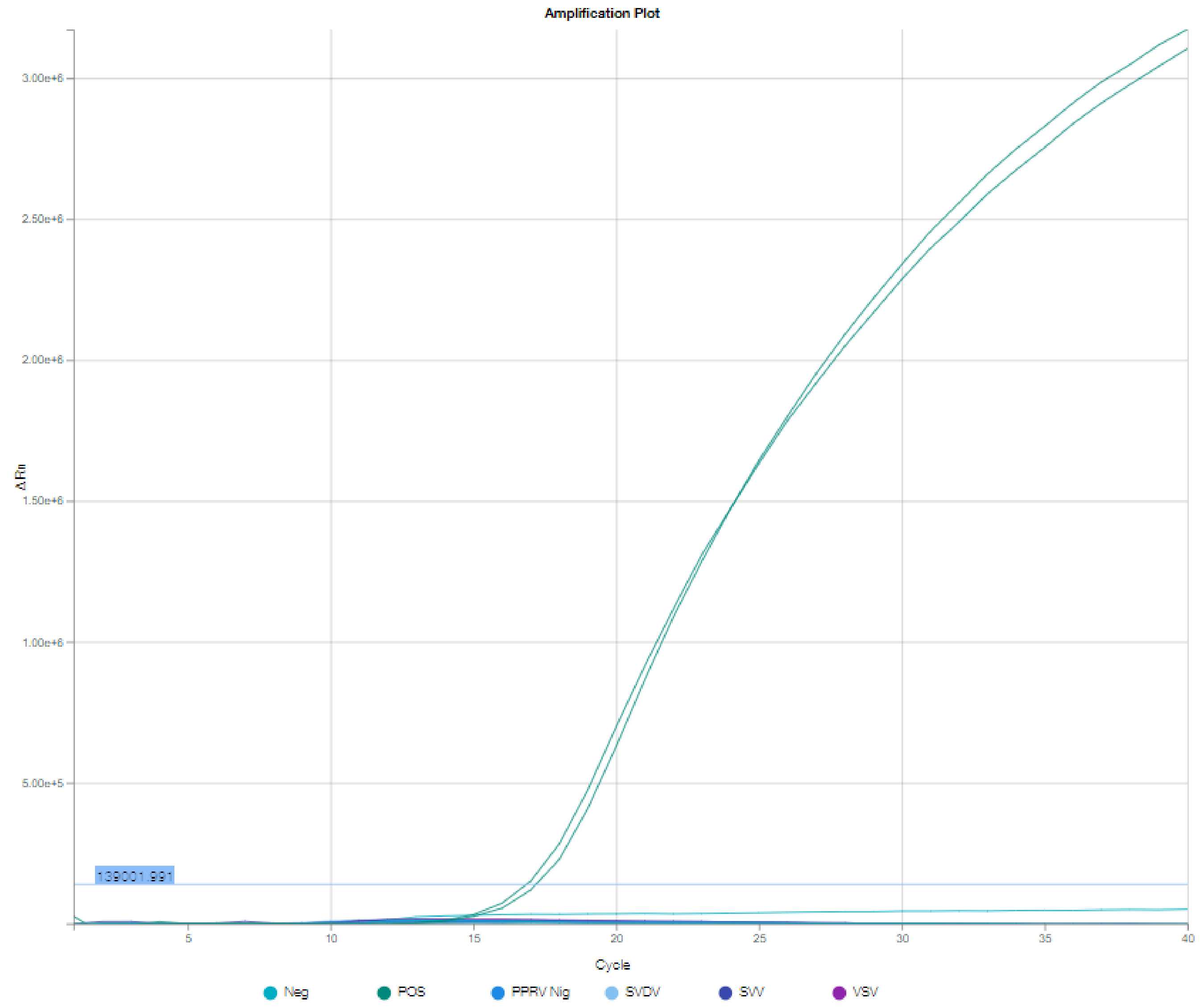
Amplification Plot
3.00e+6
2.50e+6
2.00e+6
1.50e+6
1.00e+6
5.00e+5
ΔRn
139001.991
5
10
15
20
25
30
35
40
Cycle
Neg
POS
PPRV Nig
SVDV
SVV
VSV

Supplementary Figure S3. Showing XF FMD assays spiked with high titre extracted RNA from either SVDV, VSV, SVV or PPRV, in comparison to FMDV samples and negative controls. Only the FMDV sample demonstrated amplification.

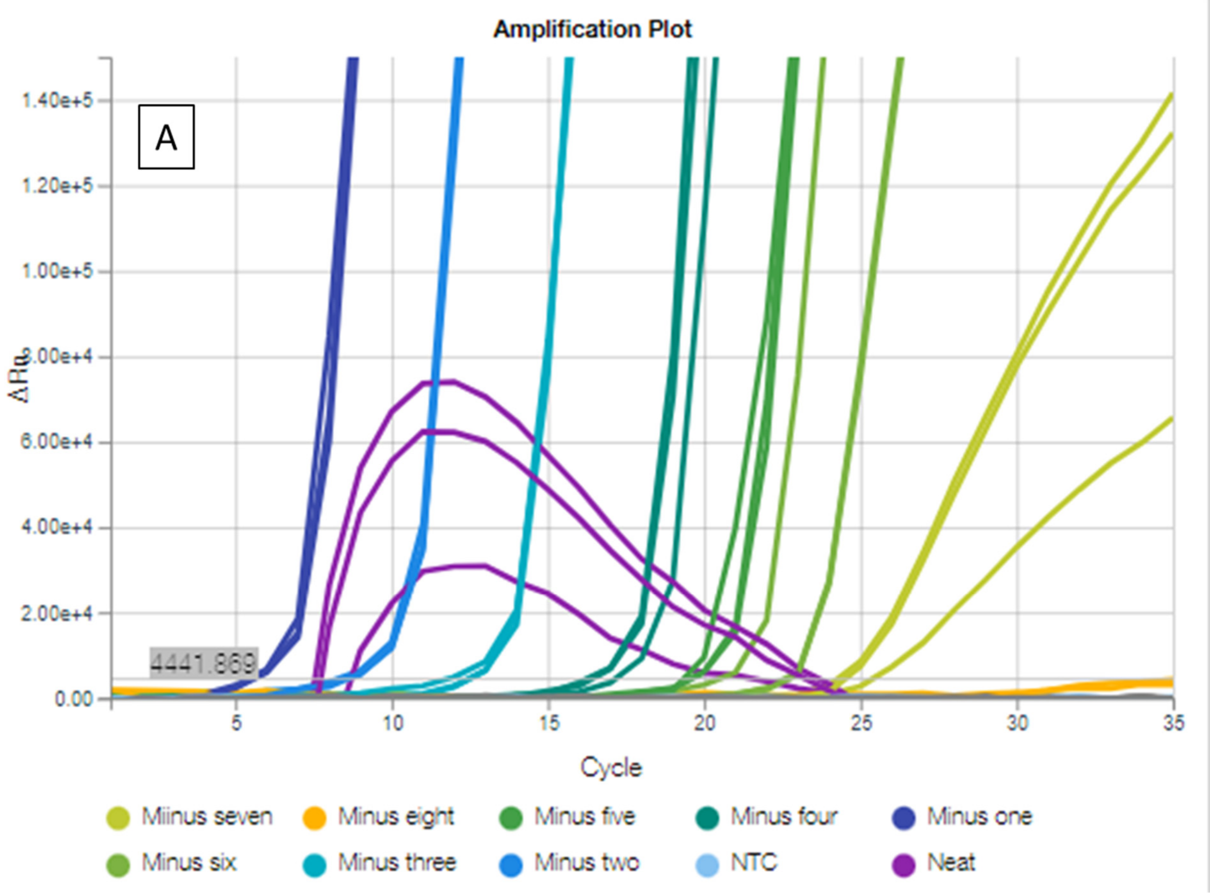

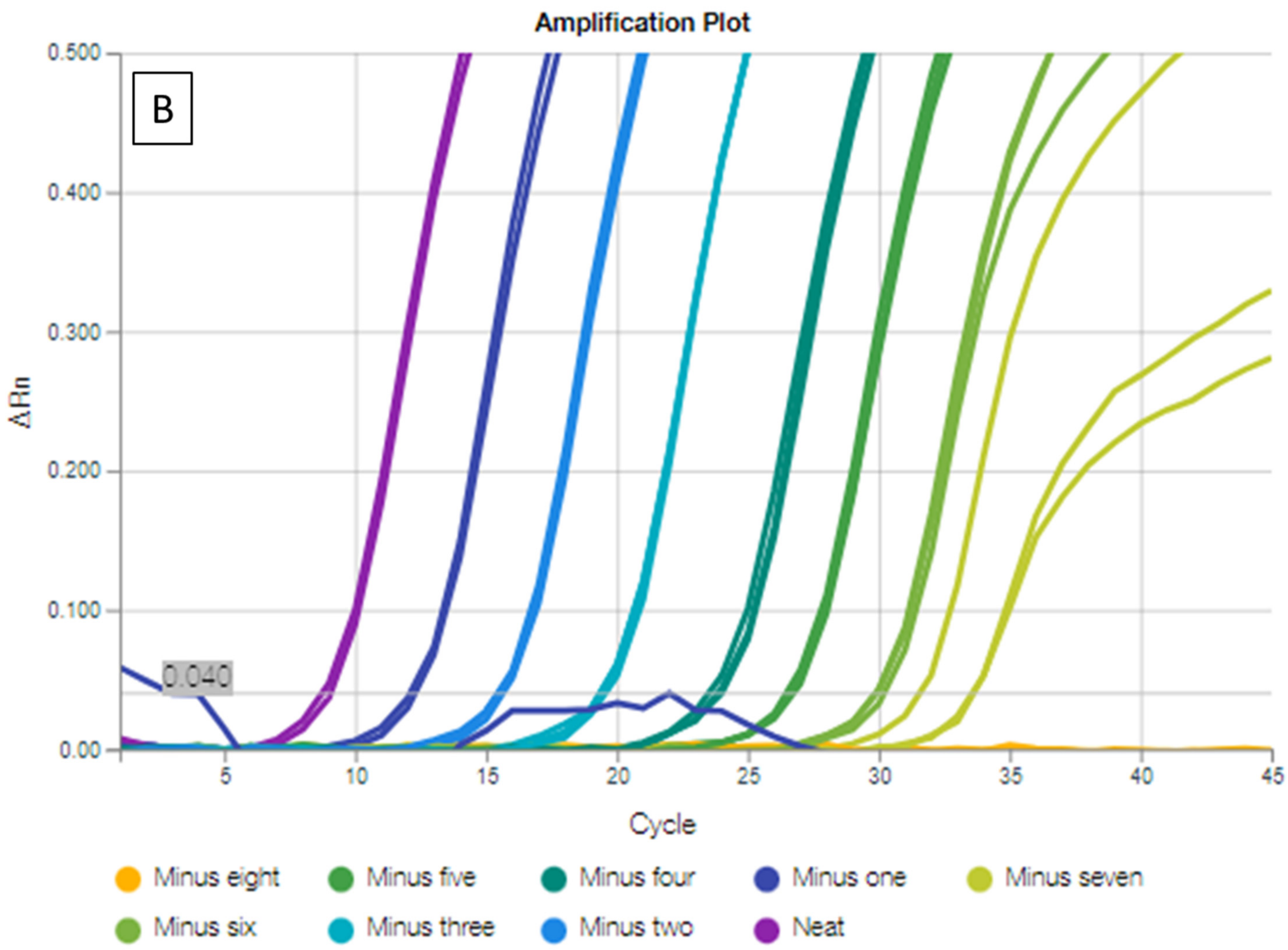

Supplementary Figure S4. Comparison of the same serial dilution of PPRV RNA extracted from a viral suspension (Sungri) when analysed using the XF assay (A) and the reference Batten assay (B). Both sets of curves use the same colour coding for the serial dilutions.

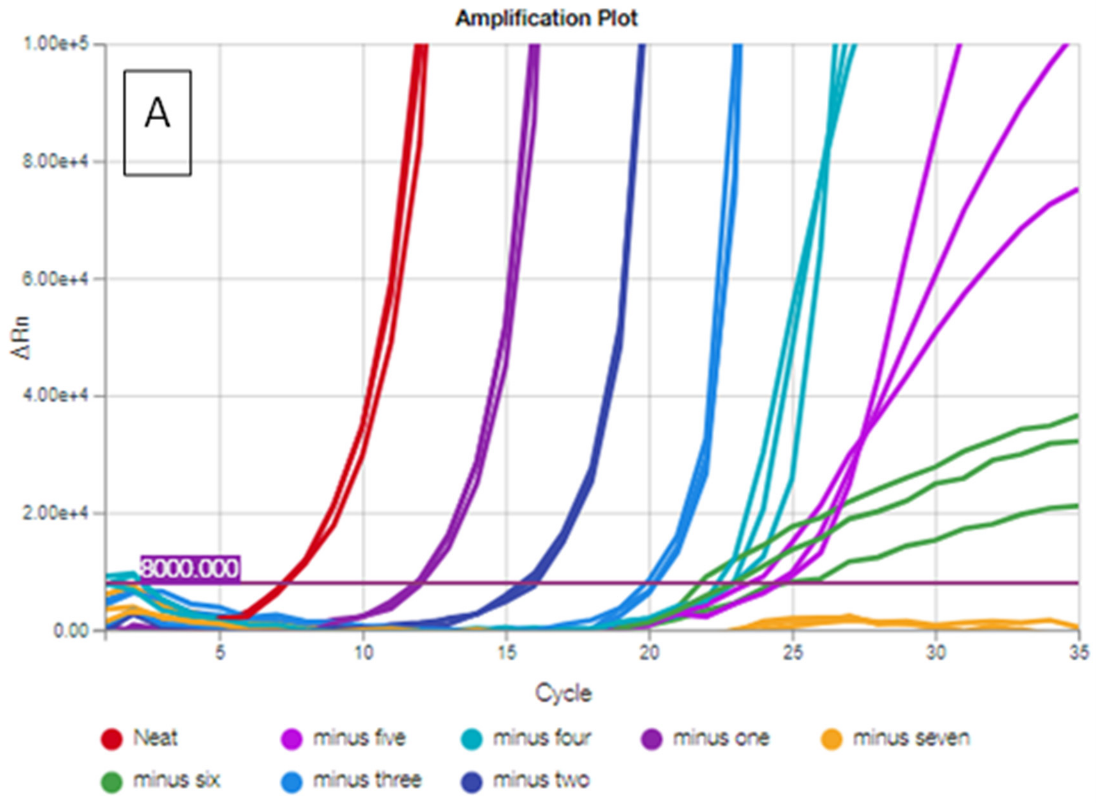
Amplification Plot
A
1.00e+5
8.00e+4
6.00e+4
4.00e+4
2.00e+4
0.00
ΔRn
8000.000
5
10
15
20
25
30
35
Cycle
Neat
minus five
minus four
minus one
minus seven
minus six
minus three
minus two

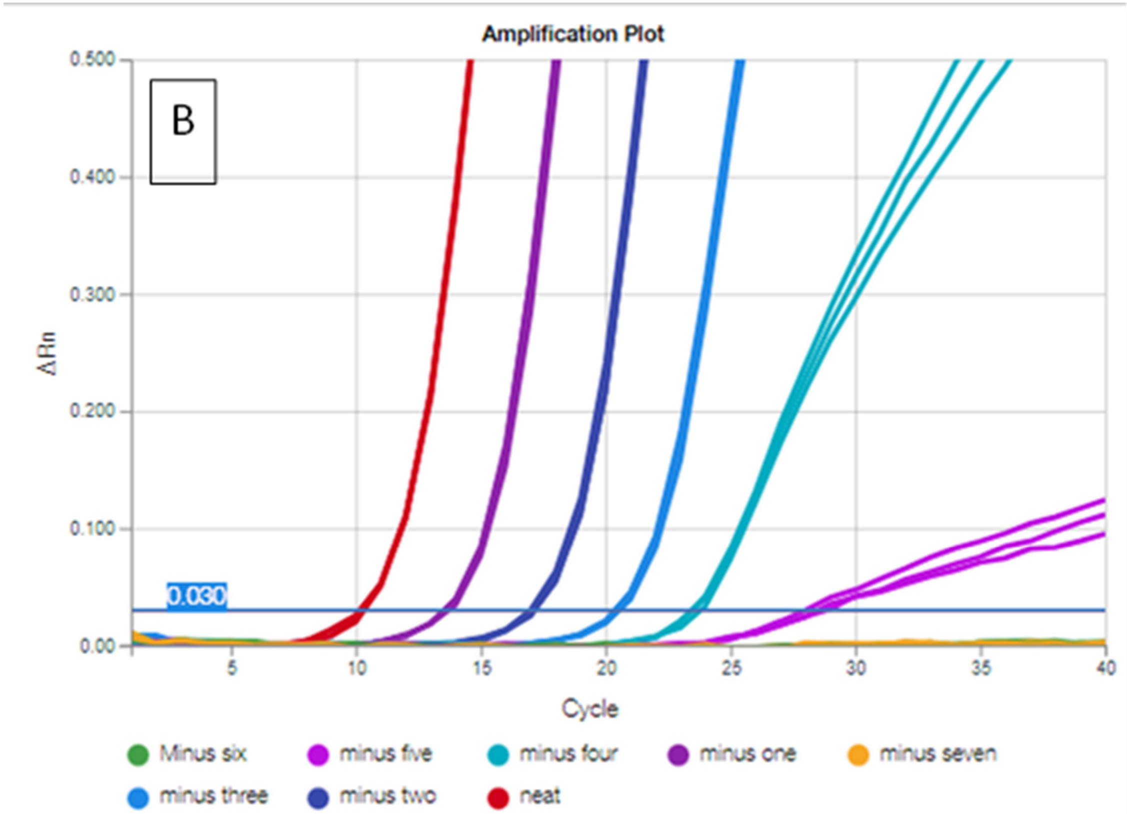

Supplementary Figure S5. Comparison of the relative performance of the XF assay (A) with the Callahan reference assay (B) using the same serially diluted RNA extracted from a viral suspension (SAT-2). Both sets of curves use the same colour coding for the serial dilutions.

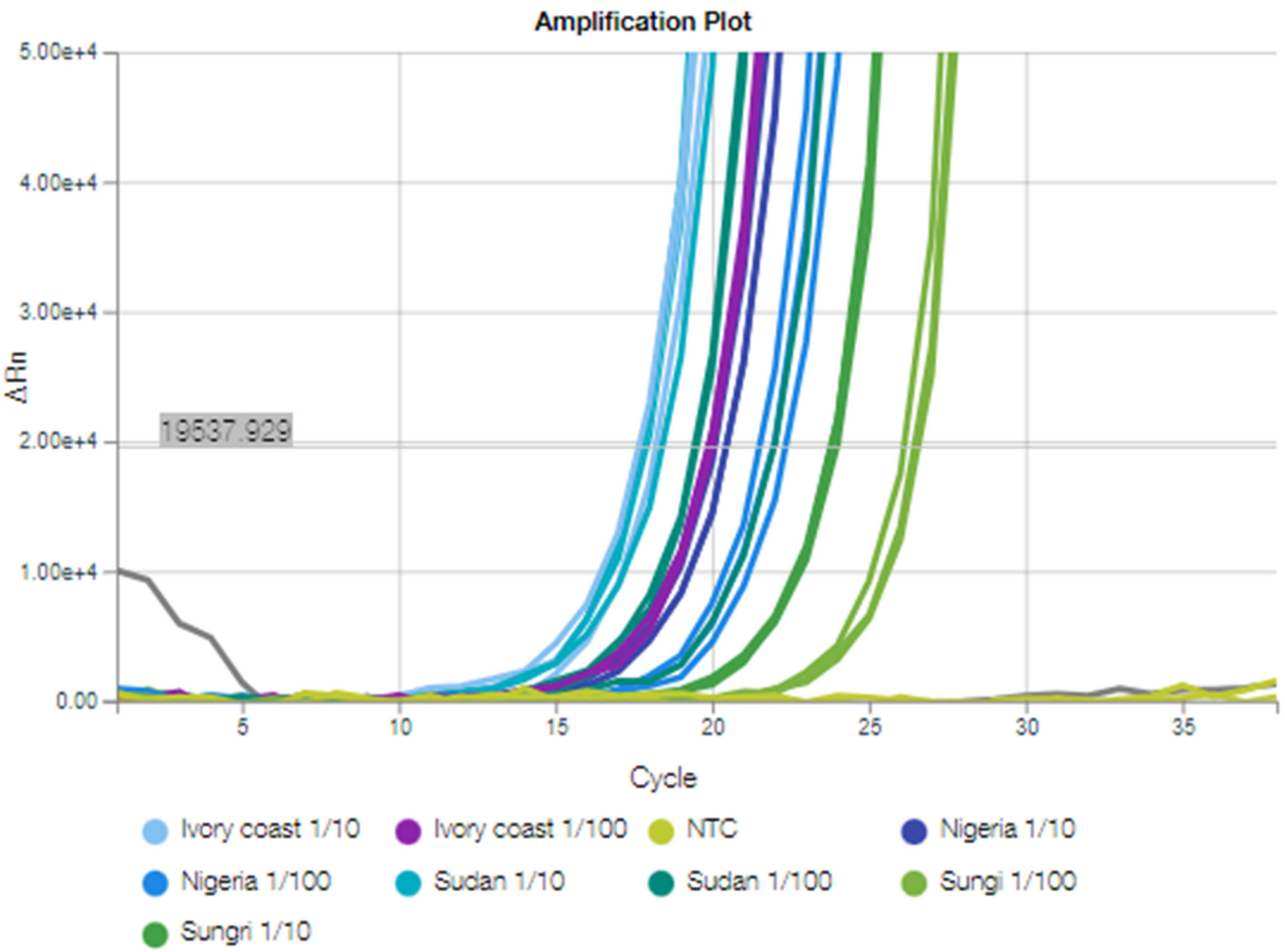

Supplementary Figure S6. Demonstration of the result of the addition of unprocessed live viral PPRV culture into an XF PPR assay. There are two serial dilutions for each of the 4 lineages (Ivory Coast, Nigeria, Sudan and Sungri) and each is shown in triplicate reactions alongside a set of NTC reactions.

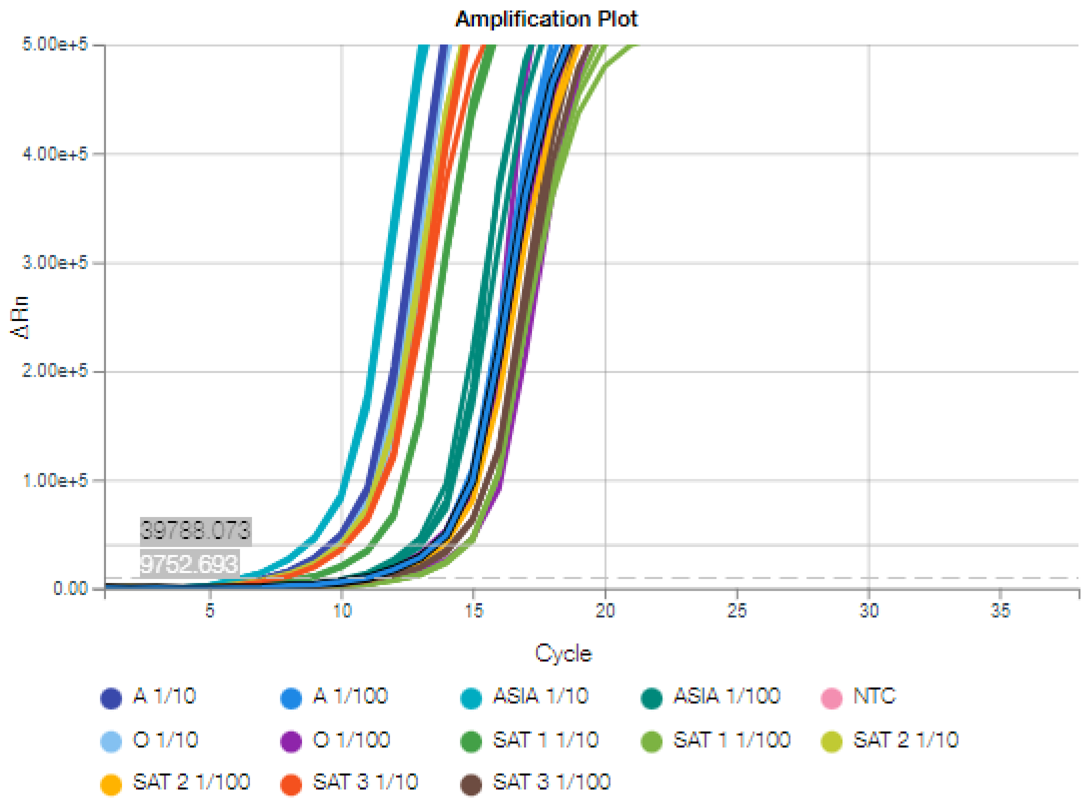

Supplementary Figure S7. Demonstration of the result of the addition of unprocessed live viral FMDV culture into an XF FMD assay. There are two serial dilutions for each of the 6 serotypes indicated in triplicate with NTC and the serotypes tested were A, Asia, O, SAT 1, SAT 2 and SAT 3.

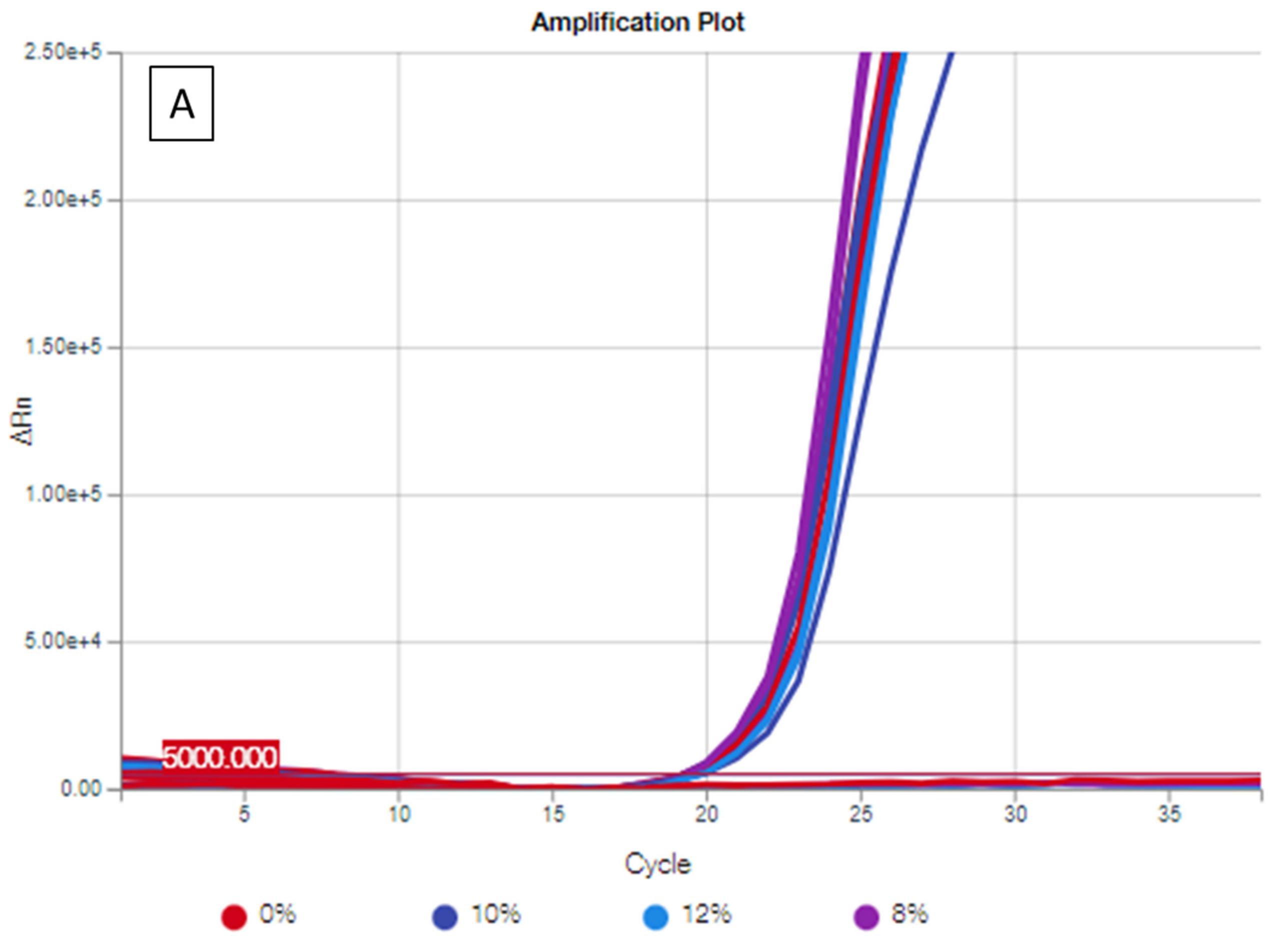

Amplification Plot
A
2.50e+5
2.00e+5
1.50e+5
1.00e+5
5.00e+4
0.00
ΔRn
5000.000
5
10
15
20
25
30
35
Cycle
0%
10%
12%
8%

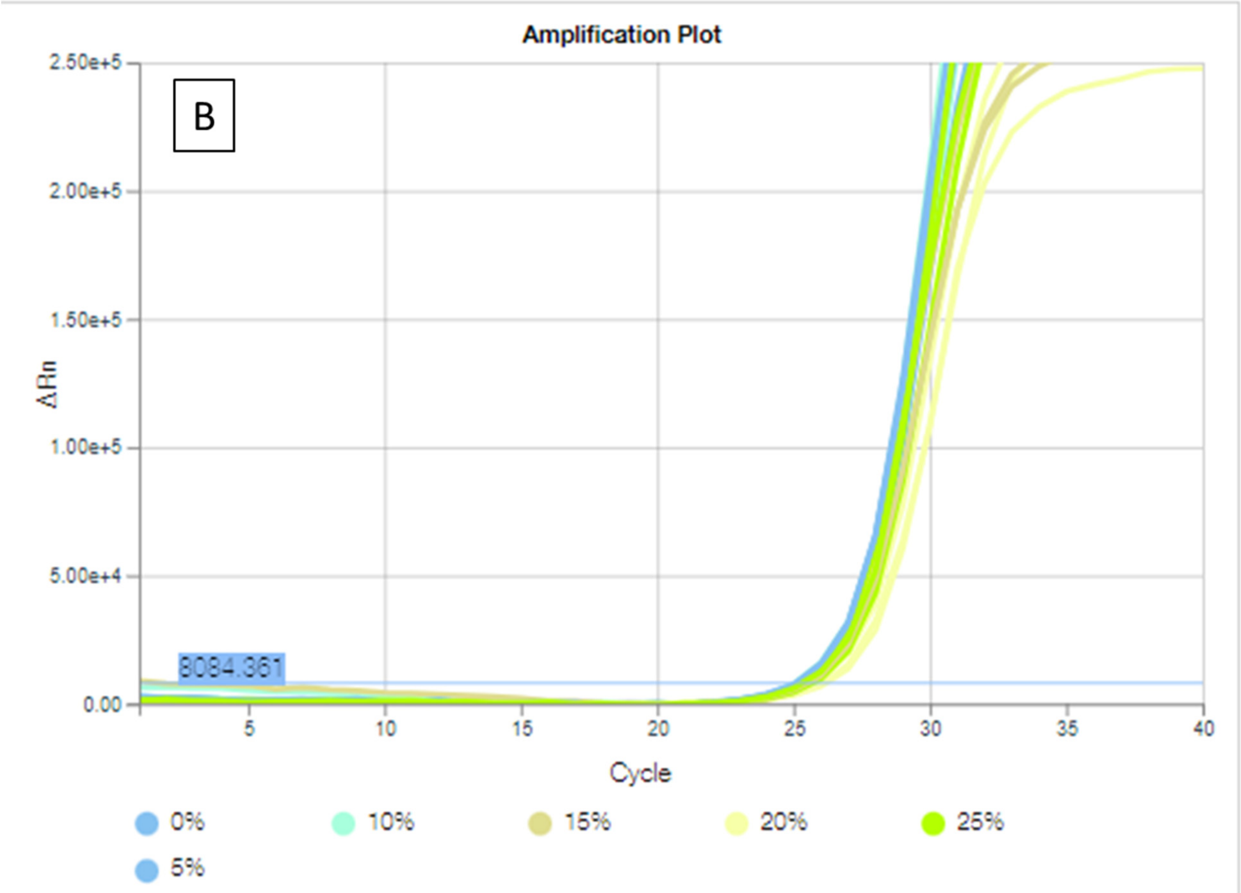

Supplementary Figure S8. Demonstration of the impact on Ct upon the addition of 0%, 8%, 10%, 12% goat nasal swab eluate on the PPR XF assay (A) and the impact on Ct of the addition of 0%, 5%, 10%, 15%, 20% and 25% cattle nasal swab eluate on a direct XF reagent assay (B). Each reaction was run in triplicate with the addition of either 1µL of unquantified PPRV genomic RNA extracted from viral culture (A) or 50 accuplex virions containing the Ebola GP/NP gene sequences (B).
